# Longitudinal changes in body mass index, height, and weight in children with acute myeloid leukemia

**DOI:** 10.1186/s12887-024-04740-z

**Published:** 2024-04-30

**Authors:** Xiaojia Wen, Hongbo He, Ruidong Zhang, Ying Wu, Yuanyuan Zhang, Wei Lin, Jiaole Yu, Jia Fan, Pengli Huang, Jiajia Chen, Wenjing Li, Chunxiu Gong, Huyong Zheng

**Affiliations:** 1 Leukemia Department, Hematology Center, Beijing Key Laboratory of Pediatric Hematology Oncology, National Key Clinical Discipline of Pediatric Hematology, National Key Discipline of Pediatrics (Capital Medical University), Key Laboratory of Major Diseases in Children, Ministry of Education, Beijing Children’s Hospital, Capital Medical University, National Center for Children’s, 56 Nanlishi Road, Beijing, 100045 China; 2 Department of Endocrinology, Genetics and Metabolism, Beijing Children’s Hospital, Capital Medical University, National Center for Children’s Health, Beijing, 100045 China

**Keywords:** Acute myeloid leukemia, Children, Body mass index, Height, Weight, Bone age

## Abstract

**Background:**

This study reported height prediction and longitudinal growth changes in Chinese pediatric patients with acute myeloid leukemia (AML) during and after treatment and their associations with outcomes.

**Methods:**

Changes in 88 children with AML in percentages according to the growth percentile curve for Chinese boys/girls aged 2–18/0–2 years for body mass index (BMI), height, and weight from the time of diagnosis to 2 years off therapy were evaluated. The outcomes of AML were compared among patients with different BMI levels.

**Results:**

The proportion of underweight children (weight < 5th percentile) increased significantly from the initial diagnosis to the end of consolidation treatment. The proportion of patients with low BMI (BMI < 5th percentile) was highest (23.08%) during the consolidation phase, and no children were underweight, but 20% were overweight (BMI > 75th percentile) after 2 years of drug withdrawal. Unhealthy BMI at the initial diagnosis and during intensive chemotherapy leads to poorer outcomes. For height, all patients were in the range of genetic height predicted based on their parents’ height at final follow-up.

**Conclusions:**

Physicians should pay more attention to the changes in height and weight of children with AML at these crucial treatment stages and intervene in time.

**Supplementary Information:**

The online version contains supplementary material available at 10.1186/s12887-024-04740-z.

## Introduction

Leukemia is the most common type of cancer in children (0–14 years old). In China, the incidence of childhood leukemia is 42.33 per 1 million [1]. Of these, acute lymphoblastic leukemia (ALL) accounts for more than 70% of all acute leukemias in children. Although acute myeloid leukemia (AML) accounts for approximately only 25% of all pediatric acute leukemias, it is the second most common cancer type in children aged 5 years and older and in adolescents (aged 15–19 years) [1]. In recent years, treatment outcomes for AML have improved due to precision risk stratification and optimized chemotherapy regimens, with overall survival reaching approximately 70% [2, 3]. At present, an increasing number of studies have confirmed the link between the nutritional status of children at the initial diagnosis of acute leukemia and leukemia relapse and disease-related death [4–9]. In addition, children are a special group, and we should not only focus on the issue of the length of their survival; the quality of life after cure is equally important. A significantly increased risk of obesity [10, 11] and reductions in linear growth and final adult height [12, 13] are common complications in cured children with ALL. However, studies of longitudinal growth changes among AML patients are rare [6]. Malnutrition, obesity, and short stature seriously affect children’s physical and mental health [11]. Therefore, it is imperative to pay more attention to the changes in body mass index (BMI), height and weight of children with AML during and after treatment and intervene in time.

In this study, we enrolled 88 patients in the Chinese Children’s Leukemia Group (CCLG)–AML2015 protocol at Beijing Children’s Hospital and evaluated BMI, weight, height and longitudinal changes in these measurements during and after therapy, along with the bone ages of patients and height of patients’ parents, to better predict the height of children. In addition, we analyzed the associations between leukemia outcomes and nutritional status at initial diagnosis and during treatment.

## Materials and methods

### Patients and the treatment protocol

Children and adolescents with AML (aged 1–18 years at the time of diagnosis) were enrolled in the CCLG-AML 2015 protocol at Beijing Children’s Hospital between 2015 and 2019. Patients who died, lacked height and weight data or underwent hematopoietic stem cell transplantation (HSCT) were censored at the time of the event. In total, 88 patients were enrolled in this study and received the CCLG-AML 2015 treatment protocol. Patient characteristics were recorded, including sex, age at the time of diagnosis, risk stratification, white blood cell counts (WBC) at the time of diagnosis, gene abnormalities, remission status, and outcomes. Remission status was evaluated by morphology after each induction therapy. Complete remission (CR), partial remission (PR) and nonremission (NR) were defined as < 5%, 5%∼20% and > 20% of undifferentiated cells in bone marrow (BM), respectively. Relapse was defined as the reappearance of leukemic cells in BM (> 5% blasts) after achieving CR [14, 15]. The stratification criteria and details of the CCLG-AML 2015 treatment protocol were published in a previous study [16]. The study was approved by the Beijing Children’s Hospital ethics committee. Written informed consent was signed by the guardians of each patient.

### BMI, weight, height

Weight and height data were obtained at the beginning of diagnosis and the end of induction therapy courses (EOI) 1 and EOI 2 and consolidation therapy course, the start of maintenance therapy, at off therapy, and at 0.5, 1, and 2 years off therapy. Patients who died, underwent HSCT or withdrew from the study were excluded for weight and height data at the time of the event. For patients aged > 2 years, we divided height, weight and BMI status into 5 levels according to the height and weight percentile curve for Chinese boys/girls aged 2–18 years and the BMI percentile curve for Chinese boys/girls aged 2–18 years. For patients aged < 2 years, we divided height and weight status into 5 levels according to the percentile curve of body length and weight for Chinese boys/girls aged 0–2 years. Level 1 belongs to 0%~5%; Level 2 belongs to 6%~25%; Level 3 belongs to 26%~75%; Level 4 belongs to 76%~95%; and Level 5 is beyond 95% [17, 18]. It was classified as low BMI if the BMI was < 5% and as overweight if the BMI was > 75%. In addition, underweight was defined as weight<5% of the percentile curve [19, 20]. To predict the genetic height of the patients, we also recorded the height of the patients’ parents at the end of the follow-up and calculated the range of genetic height of patients according to the method described in a previous study [21].

### X-ray examination of bone age

Patients did X-ray examinations of left wrist, and the imaging physicians reported the patient’s specific bone age by Greulich and Pyle method. X-ray examination of bone age data was obtained at the beginning of diagnosis and consolidation therapy course, at the start of maintenance therapy, at off therapy, and at 0.5, 1, and 2 years off therapy. In this study, accelerated bone maturation was considered if the bone age was 1–2 years older than the chronological age. If the bone age was less than the chronological age of 1–2 years, delayed bone maturation was considered [22].

### Statistical analysis

The reference date for concluding data collection for statistical analysis was set as October 31, 2021. The Mann‒Whitney U test for continuous variables, chi-squared test and Fisher’s exact test for categorical variables were used to compare the clinical characteristics of patients between the low BMI and non-low BMI groups. Chi-squared test and Fisher’s exact test for categorical variables were used to compare patients’ BMI, height and weight among different time points of treatment. Overall survival (OS) was defined as the time from diagnosis to death from any cause or to last follow-up. Relapse-free survival (RFS) was defined as the time between diagnosis and the first relapse. Event-free survival (EFS) was defined as the time from diagnosis to last follow-up or the first event. Events were defined as relapse, death from any cause, or secondary malignancy. EFS, RFS and OS were estimated by the Kaplan‒Meier method and compared using the log-rank test. All tests were two-tailed, and *P* < 0.05 was considered statistically significant. SPSS 26.0 software (SPSS Inc., Chicago, IL) was used for all statistical analyses.

## Results

### Patient clinical characteristics according to nutritional status

A total of 88 patients were included in this study. None of the patients received tyrosine kinase inhibitors (TKI) or underwent radiotherapy as part of their treatment. Additionally, none of the patients were found to have comorbidities related to weight or height, such as trisomy 21. Their clinical characteristics according to BMI status and the number of patients at each time point are summarized in Table 1. Sex, age at diagnosis, WBC at diagnosis, and genetic abnormalities did not show significant differences between the low BMI and non-low BMI groups. In addition, the chemotherapy process and disease remission status also showed no significant differences between the low BMI and non-low BMI groups, including EOI 1, EOI 2, relapse after CR, events, and death.

**Table 1:** Patient clinical characteristics between the low BMI and non-low BMI groups

Clinical characteristic	Low BMI group (*n* = 11)	Non-low BMI group (*n* = 77)	P value
Sex			0.743
Male	7 (63.64)	45 (58.44)	
Female	4 (36.36)	32 (41.56)	
Age at diagnosis (years)	6.25 (1.92–12.83)	6.67 (0.58–15.58)	0.472
WBC at diagnosis(×10^9^/L)	12.69 (2–45)	12.99 (1-138)	0.334
Fusion transcripts			0.341
Without known genes	3 (27.27)	31 (40.26)	
*RUNX1-RUNX1T1*	3 (27.27)	24 (31.17)	
*KMT2A- KMT2AT3*	2 (18.18)	4 (5.19)	
*KMT2A-others*	1 (18.18)	9 (11.69)	
*CBFβ-MYH11*	1 (18.18)	6 (7.79)	
*FUS-ERG*	0 (0)	2 (2.60)	
*NUP98-HOXA9*	1 (18.18)	1 (1.30)	
*KIT* mutation			0.350
Positive	0 (0)	10 (12.99)	
Negative	11 (100)	67 (87.01)	
EOI 1 status, *n* = 87^#^			0.484
CR	8 (72.73)	56 (72.73)	
PR	3 (27.27)	12 (15.58)	
NR	0 (0)	8 (10.39)	
EOI 2 status, *n* = 83^@^			0.692
CR	11 (100)	62 (80.52)	
PR	0 (0)	8 (10.39)	
NR	0 (0)	2 (2.60)	
Risk rank at initial diagnosis^*^, *n* = 86^$^			0.182
SR	5 (45.45)	18 (23.38)	
MR	3 (27.27)	15 (19.48)	
HR	3 (27.27)	42 (54.55)	
Relapsed after CR			0.701
No	8 (72.72)	60 (77.92)	
Yes	3 (27.27)	17 (22.08)	
Event occurred			0.448
No	6 (54.55)	51 (66.23)	
Yes	5 (45.45)	26 (33.77)	
Death			0.915
No	9 (81.82)	64 (83.12)	
Yes	2 (18.18)	13 (16.88)	

*Note* Normal data are given as medians (ranges); qualitative variables are given as numbers (percentages). *EOI* the end of induction therapy courses. ^#^one patient did not undergo bone marrow biopsy after induction 1; ^@^ three patients did not continue induction 2, and two patients did not undergo bone marrow biopsy after induction 2; ^$^two patients did not continue chemotherapy after induction 1. ^*^ Patients were excluded if they underwent HSCT after chemotherapy, and risk rank was defined only as the initial diagnosis. Low-BMI was defined as BMI less than 5% at initial diagnosis, induction or consolidation chemotherapy phases

### BMI, height and weight among different chemotherapy processes

**Fig. 1:**
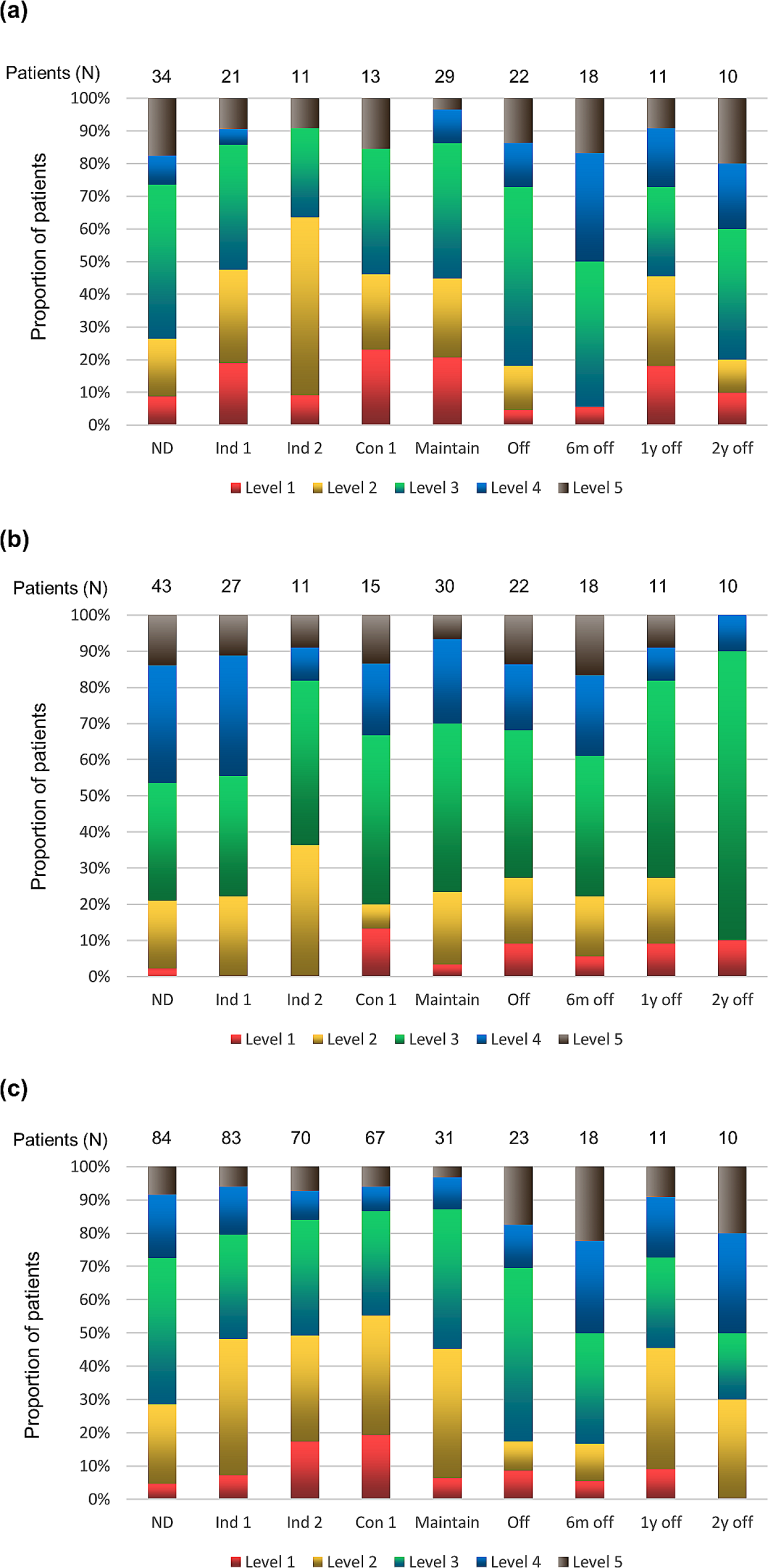
Proportion of patients by BMI (**a**), height (**b**), and weight (**c**) at different treatment stages. *Note ND* initial diagnosis; *Ind* induction; *Con* consolidation; *off* withdrawal of drugs; *m* month; *y* year. According to height, weight and BMI percentile curve for Chinese boys/girls aged 0–18, level 1: 0%~5%; level 2: 6%∼25%; level 3: 26%∼75%; level 4: 76%∼95%; level 5: beyond 95%. The number above the bar graph represented the exact number evaluated for each parameter at each assessment point

For BMI, the proportion of patients with BMI at levels 1 to 2 increased from initial diagnosis to EOI 2. During the consolidation phase, the proportion of patients with a BMI of level 1 was the highest (23.08%) in comparison with the other treatment phases. However, compared with the other treatment phases, the proportion of patients with a BMI of level 1 was lowest (4.55%) in the off-therapy phase (Fig. 1a). There was no significant difference in BMI between the different treatment stages compared with BMI at initial diagnosis (Table 2a). For height, only the change in the two-year off-treatment was significantly different from the initial time of diagnosis, and 80% of patients were in growth status level 3 (*P* = 0.033). (Fig. 1b, Table 2b). For weight, the proportion of underweight children (level 1) increased from 4.76 to 19.4% from initial diagnosis to the end of consolidation treatment (Table 2c). In addition, the proportion of patients with weight < 5% or belonging to the 6%∼25% percentile curve increased in the induction 2 and consolidation phases compared with the initial diagnosis, with *P* values of 0.034 and 0.007, respectively (Table 2c). The proportion of underweight children did not change significantly from the maintenance period to 1 year after drug withdrawal. Interestingly, after 2 years of drug withdrawal, there were no underweight children (level 1), but 20% of patients were overweight at level 5 (Fig. 1c).

**Table 2a:** Comparison of BMI at the time of initial diagnosis with other periods of treatment

Treatment Stages	level 1 (n, %)	level 2 (n, %)	level 3 (n, %)	level 4 (n, %)	level 5 (n, %)	χ^2^	P value
ND (*n* = 34)	3 (8.82)	6 (17.65)	16 (47.06)	3 (8.82)	6 (17.65)		
Ind 1^*^ (*n* = 21)	4 (19.05)	6 (28.57)	8 (38.10)	1 (4.76)	2 (9.52)	2.899	0.575
Ind 2^*^ (*n* = 11)	1 (9.09)	6 (54.55)	3 (27.27)	0 (0)	1 (9.09)	/	0.204
Con 1^*^ (*n* = 13)	3 (23.08)	3 (23.08)	5 (38.46)	0 (0)	2 (15.38)	/	0.669
Maintain^*^ (*n* = 29)	6 (20.69)	7 (24.14)	12 (41.38)	3 (10.34)	1 (3.45)	5.235	0.294
Off^*^ (*n* = 22)	1 (4.55)	3 (13.64)	12 (54.55)	3 (13.64)	3 (13.64)	1.067	0.899
6 m off^*^ (*n* = 18)	1 (5.56)	0 (0)	8 (44.44)	6 (33.33)	3 (16.67)	/	0.117
1y off^*^ (*n* = 11)	2 (18.18)	3 (27.27)	3 (27.27)	2 (18.18)	1 (9.09)	2.820	0.588
2y off^*^ (*n* = 10)	1 (10)	1 (10)	4 (40)	2 (20)	2 (20)	1.181	0.881

*Note ND* initial diagnosis; *Ind* induction; *Con* consolidation; off: withdrawal of drugs; *m* month; *y* year. According to height, weight and BMI percentile curve for Chinese boys/girls aged 0–18, level 1: 0%~5%; level 2: 6%~25%; level 3: 26%~75%; level 4: 76%~95%; level 5: beyond 95%. ^*^Compared with ND

**Table 2b:** Comparison of height at the time of initial diagnosis with other periods of treatment

Treatment Stages	level 1 (n, %)	level 2 (n, %)	level 3 (n, %)	level 4 (n, %)	level 5 (n, %)	χ^2^	P value
ND (*n* = 43)	1 (2.33)	8 (18.6)	14 (32.56)	14 (32.56)	6 (13.95)		
Ind 1^*^ (*n* = 27)	0 (0)	6 (22.22)	9 (33.33)	9 (33.33)	3 (11.11)	/	0.250
Ind 2^*^ (*n* = 11)	0 (0)	4 (36.36)	5 (45.45)	1 (9.09)	1 (9.09)	/	0.418
Con 1^*^ (*n* = 15)	2 (13.33)	1 (6.67)	7 (46.67)	3 (20)	2 (13.33)	4.634	0.394
Maintain^*^ (*n* = 30)	1 (3.33)	6 (20)	14 (46.67)	7 (23.33)	2 (6.67)	2.431	0.657
Off^*^ (*n* = 22)	2 (9.09)	4 (18.18)	9 (40.91)	4 (18.18)	3 (13.64)	2.790	0.590
6 m off^*^ (*n* = 18)	1 (5.56)	3 (16.67)	7 (38.89)	4 (22.22)	3 (16.67)	1.086	0.896
1y off^*^ (*n* = 11)	1 (9.09)	2 (18.18)	6 (54.55)	1 (9.09)	1 (9.09)	4.289	0.368
2y off^*^ (*n* = 10)	1 (10)	0 (0)	8 (80)	1 (10)	0 (0)	/	0.033

*Note ND* initial diagnosis; *Ind* induction; *Con* consolidation; off: withdrawal of drugs; *m* month; *y* year. According to height, weight and BMI percentile curve for Chinese boys/girls aged 0–18, level 1: 0%~5%; level 2: 6%~25%; level 3: 26%~75%; level 4: 76%~95%; level 5: beyond 95%. ^*^Compared with ND

**Table 2c:** Comparison of weight at the time of initial diagnosis with other periods of treatment

Treatment Stages	level 1 (n, %)	level 2 (n, %)	level 3 (n, %)	level 4 (n, %)	level 5 (n, %)	*χ* ^*2*^	*P* value
ND (n = 84)	4 (4.76)	20 (23.81)	37 (44.05)	16 (19.05)	7 (8.33)		
Ind 1^*^ (n = 83)	6 (7.23)	34 (40.96)	26 (31.33)	12 (14.46)	5 (6.02)	6.907	0.154
Ind 2^*^ (n = 70)	12 (17.14)	22 (31.43)	24 (34.29)	6 (8.57)	5 (7.14)	10.396	0.034
Con 1^*^ (n = 67)	13 (19.4)	24 (35.82)	21 (31.34)	5 (7.46)	4 (5.97)	14.823	0.007
Maintain^*^ (n = 31)	2 (6.45)	12 (38.71)	13 (41.94)	3 (9.68)	1 (3.23)	4.164	0.384
Off^*^ (n = 23)	2 (8.7)	2 (8.7)	12 (52.17)	3 (13.04)	4 (17.39)	4.786	0.310
6 m off^*^ (n = 18)	1 (5.56)	2 (11.11)	6 (33.33)	5 (27.78)	4 (22.22)	4.428	0.351
1y off^*^ (n = 11)	1 (9.09)	4 (36.36)	3 (27.27)	2 (18.18)	1 (9.09)	1.158	0.813
2y off^*^ (n = 10)	0 (0)	3 (30)	2 (20)	3 (30)	2 (20)	/	0.350

Note: ND: initial diagnosis; Ind, induction; Con, consolidation; off: withdrawal of drugs; m, month; y, year. According to height, weight and BMI percentile curve for Chinese boys/girls aged 0–18, level 1: 0%~5%; level 2: 6%~25%; level 3: 26%~75%; level 4: 76%~95%; level 5: beyond 95%. ^*^Compared with ND

### Outcomes according to nutritional status

**Fig. 2:**
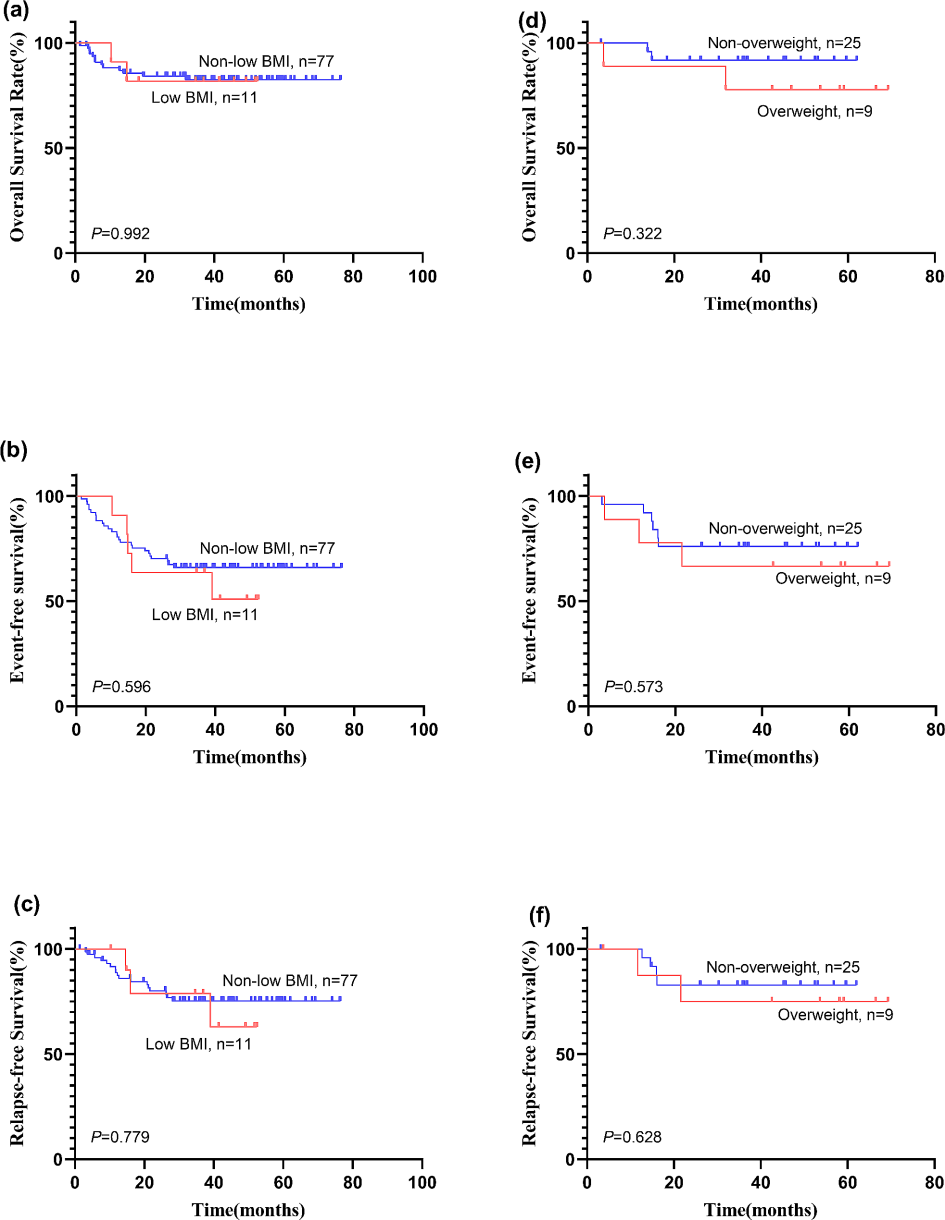
Patient outcomes according to nutritional status. 3-year overall survival (**a**) (**d**), 3-year event-free survival (**b**) (**e**) and 3-year relapse-free survival (**c**) (**f**) of AML patients according to different nutritional statuses. Low BMI was classified if the BMI was < 5% and overweight if the BMI was > 75%

At the median follow-up of 37.1 months, the 3-year EFS and OS of all patients were 63.9 ± 5.2% and 84.2 ± 4.2%, respectively. In the initial diagnosis, induction and consolidation chemotherapy phases, patients with low BMI showed a lower 3-year EFS (50.9 ± 16.3% vs. 66 ± 5.4%) than patients without low BMI (*P* = 0.596). Similarly, the 3-year RFS was also lower in the low BMI group (63 ± 17.7% vs. 75.3 ± 5.2%) than in the non-low BMI group (*P* = 0.779). The 3-year OS showed no significant difference between the low BMI and non-low BMI groups (82.4 ± 4.4% vs. 81.8 ± 11.6%), *P* = 0.992 (Fig. 2a-c).

Furthermore, patients were categorized into non-overweight and overweight groups according to BMI at initial diagnosis. Analysis of clinical characteristics at the onset revealed no statistically significant differences between the overweight and non-overweight group (Table S1). Compared to the nonoverweight patients, the overweight patients showed a lower 3-year OS (77.8 ± 13.9% vs. 91.7 ± 5.6%), *P* = 0.322 (Fig. 2d). The overweight patients showed a lower 3-year EFS (66.7 ± 15.7% vs. 76 ± 8.5%) than the nonoverweight patients (*P* = 0.573) (Fig. 2e). Similarly, the 3-year RFS was also lower in the overweight group (75 ± 15.3% vs. 82.9 ± 7.8%) than in the nonoverweight group (*P* = 0.628) (Fig. 2f). Furthermore, we also divided patients’ BMI at ND into low BMI, normal BMI and high BMI (overweight). The results showed that normal BMI at ND patients had superior outcomes compared with low BMI and high BMI patients, details in Figure S1.

### Imaging X-ray examination of bone age during different treatment stages

We divided the patients into two groups: during medication and after withdrawal of medication. There were a total of sixty-four and forty-six assessments before and after drug discontinuation, respectively. There was no statistically significant difference between bone age and chronological age at the initial diagnosis, consolidation and maintenance treatment phases (*P* = 0.576) (Figure S2a). Furthermore, there was no statistically significant difference between bone age and chronological age at drug withdrawal, six months, one year, or two years (Figure S2b-e). In addition, we analyzed the ratio of the individual values between the different treatment processes. There was no significant difference between the initial diagnosis and continuous treatment phases. Interestingly, the only difference between initial diagnosis and consolidation was a borderline *P* value of 0.066, and 100% of the patients’ bone ages were normal (level 1 and level 2) after consolidation chemotherapy (Table 3).

**Table 3:** Comparison of bone age evaluation status at the time of initial diagnosis with other periods of treatment

Treatment Stages	level 1 (n, %)	level 2 (n, %)	level 3 (n, %)	level 4 (n, %)	level 5 (n, %)	χ^2^	P value
ND (*n* = 28)	17 (60.7)	8 (28.6)	1(3.6)	2 (7.1)	0 (0)		
Con 1^*^ (*n* = 26)	23 (88.5)	3 (11.5)	0 (0)	0 (0)	0 (0)	/	0.066
Maintain* (*n* = 10)	6 (60)	1 (10)	1 (10)	2 (20)	0 (0)	/	0.312
Off^*^ (*n* = 15)	9 (60)	3 (20)	2 (13.3)	1 (6.7)	0 (0)	/	0.774
6 m off^*^ (*n* = 8)	3 (37.5)	2 (25)	2 (25)	1 (12.5)	0 (0)	/	0.310
1y off^*^ (*n* = 8)	4 (50)	2 (25)	0 (0)	1 (12.5)	1 (12.5)	4.054	0.455
2y off^*^ (*n* = 7)	6 (85.7)	0 (0)	1 (14.3)	0 (0)	0 (0)	/	0.280

*Note ND* initial diagnosis; *Con* consolidation; *off* withdrawal of drugs; *m* month; *y* year. ^*^Compared with ND. Level 1: -1 ≤ BA-CA ≤ 1; level 2: 1 < BA-CA ≤ 2; level 3: -2 ≤ BA-CA<-1; level 4: BA-CA<-2; level 5: BA-CA > 2. BA-CA was defined as bone age minus chronological age

### Estimation of genetic height for patients after chemotherapy treatment

The height data of the parents of 12 patients whose guardians were willing to provide were collected. Until the last follow-up, 5 patients were off 2 years, 1 patient was off 1 year, 2 patients were in maintenance, 3 patients were in consolidation, and 1 patient was in EOI 1. We found that the height rank of the patients was within the normal range during the treatment period, fluctuating between level 2 and level 5. At the final follow-up, 11 patients were at level 3 or above and within the range of genetic height (Figure S3). Among these 12 patients, one patient (#11) was 15.5 years old, another patient (#12) was 13.5 years old at the initial diagnosis, while the remaining 10 patients were under 12 years old. However, it is noteworthy that only patient #2 approached the lower boundary of the genetic height range at the final follow-up, with the other patients positioned in the middle or upper boundary. Of these 12 patients, accelerated bone maturation was present in only one patient (#4), and the differences between bone age and chronological age were less than 1 in the other 11 patients.

## Discussion

Chemotherapy may affect the final adult height and nutritional status of children with leukemia, which in turn may affect the physical and mental health of patients after discontinuation of the drug [11, 12]. Additionally, relatively high or low BMI may be associated with leukemia outcomes [4–7]. Therefore, we evaluated the longitudinal changes in BMI, weight and height of 88 pediatric patients with AML during and after chemotherapy. We predicted the effect of chemotherapy on the final adult height of AML patients by the bone age and height of the patients’ parents. Moreover, we explored the associations between leukemia outcomes and BMI at initial diagnosis and during treatment. To our knowledge, this is the first study to use bone age and parental height in predicting the final height of children with AML. Meanwhile, this is the first study to use the growth curves of Chinese children and adolescents to assign descriptive values to BMI, weight, and height for children with AML, which minimizes the influence of different growth rates at different ages, genders and races on the results.

In this study, we found that during leukemia treatment, patients’ weight loss was mainly concentrated during induction and consolidation therapy, whereas the short-term effect of chemotherapy on height was not significant, resulting in reduced BMI in children during induction and consolidation therapy. The changes in weight are consistent with the current published AML study [6]. After the maintenance period, the children’s weight and BMI gain were significantly accelerated, which is considered to be related to the low chemotherapy intensity and low tumor load after maintenance therapy. Over time, surprisingly, 20% of the children appeared to be overweight after two years of drug withdrawal, and we consider the high rate of overweight to be associated with chemotherapy drug effects and lack of exercise after cure. However, compared with the initial diagnosis BMI, there was no significant difference among the different treatment processes. This may be related to the fact that all children with leukemia in our hospital are equipped with a nutritious meal, narrowing the weight differences between treatment periods, or it may be related to the low sample size. Conversely, in patients with ALL, weight gain begins during induction therapy, which is the result of exposure to glucocorticoids and asparaginase, unhealthy diets, and lack of activity, and continues even after the end of therapy [23, 24]. The difference in weight change between patients with ALL and those with AML underdoing intense chemotherapy could be attributed to disparities in their chemotherapy regimens. Notably, the administration of cytarabine [25, 26] may induce stomatitis, contributing to weight loss in AML patients. Conversely, the use of glucocorticoids [27] may lead to weight gain in ALL patients. As a high BMI can put survivors at increased risk of metabolic syndrome, early nutritional intervention and appropriate daily physical activity should be implemented in children with leukemia as early as possible [28].

For height, our study found that the proportional change at each time point was not significant except at the two-year off therapy period. Height growth accelerated significantly after two years of drug withdrawal, and the majority of patients were in the 26-75% range of height for children of the same age and gender. Again, there was no significant difference between bone age and chronological age during and after treatment, and for the 12 patients who were followed up to their parents’ height, the height at last follow-up was within the range of the genetic prediction of height. However, one study [6] on children with AML found that height Z scores are significantly lower than baseline scores not only during therapy but also throughout the off-therapy period, and linear growth is further affected by HSCT. The reason for the difference in the results of the two studies may be that our study excluded AML patients who underwent HSCT.

Our study found that patients with low BMI in the initial diagnosis and induction chemotherapy stages or with overweight in the initial diagnosis showed relatively lower 3-year EFS, 3-year RFS and 3-year OS. Previous studies also demonstrated that patients with AML in unhealthy BMI categories (underweight or overweight/obese) at diagnosis had significantly worse survival and more treatment-related mortality than healthy-weight individuals [29, 30]. However, there was no significant difference in the effect of BMI on leukemia outcomes in our study. The revolutionary changes in AML treatment have marked small molecule inhibitors and biologic agents over the years [31, 32]. Some supportive care, such as antibiotic prophylaxis, transfusion support, management of hyperleukocytosis and neutropenic fever, and better education for patient families and clinical staff, have also improved [33, 34]. Patients with transplantation were excluded from this study, and most high-risk patients were excluded from the study, which would have resulted in prognostic bias. The above therapeutic advances and outcome bias due to enrollment criteria may have contributed to the outcomes being similar regardless of BMI status at diagnosis. In addition, the survival rate of children with AML in the present study was significantly higher than that of the reported studies [2, 3], which is also considered to be related to the enrollment criteria. In addition, the small sample size of this study may also affect the *P* value.

This study had some limitations. Because of death or transplantation, the number of patients declined over time. Most of the children were under 18 years of age at the time of the follow-up visit, and we had no information on whether patients had reached their final adult height. This study did not include the effects of confounding factors such as environment, diet, and exercise on height and weight. However, this study predicted the final height of children with AML by bone age and parental height and used the growth curves of Chinese children and adolescents to minimize the influence of different growth rates at different ages and sexes on the results, providing great clinical reference value.

## Conclusion

In conclusion, this study revealed longitudinal changes in BMI, weight and height during the on-therapy and off-therapy periods, predicted the final height of children with AML by bone age and parental height, and demonstrated the relationship between BMI and leukemia outcomes. AML showed significant weight loss and decreased BMI during induction therapies, a gradual increase in weight and BMI after the maintenance period, and a greatly increased risk of overweight after discontinuation of chemotherapy, but treatment did not significantly affect the height of the patients with AML. Unhealthy BMI might reduce the prognosis of pediatric AML patients, but advances in leukemia treatment and supportive care have greatly reduced the prognostic impact of BMI at the time of the initial diagnosis. It is imperative to focus on the changes in BMI, height and weight of children with AML during and after treatment and intervene in time.

### Electronic supplementary material

Below is the link to the electronic supplementary material.

Supplementary Material 1

Supplementary Material 2

Supplementary Material 3

Supplementary Material 4

## Data Availability

The data and material of this study are available from the corresponding author upon reasonable request.

## Abbreviations

ALL: acute lymphoblastic leukemia
AML: acute myeloid leukemia
BM: bone marrow
BMI: body mass index
CCLG: Chinese Children’s Leukemia Group
CR: complete remission
EFS: event-free survival
EOI: the end of induction therapy courses
HSCT: hematopoietic stem cell transplantation
NR: nonremission
OS: overall survival
PR: partial remission
RFS: relapse-free survival
TKI: tyrosine kinase inhibitors
WBC: white blood cell counts
