# Supplementary figures and images for: Longitudinal changes in body mass index, height, and weight in children with acute myeloid leukemia
Source: BMC Pediatr. 2024 Apr 30;24:293. doi: 10.1186/s12887-024-04740-z (PMC11061944; doi:10.1186/s12887-024-04740-z)

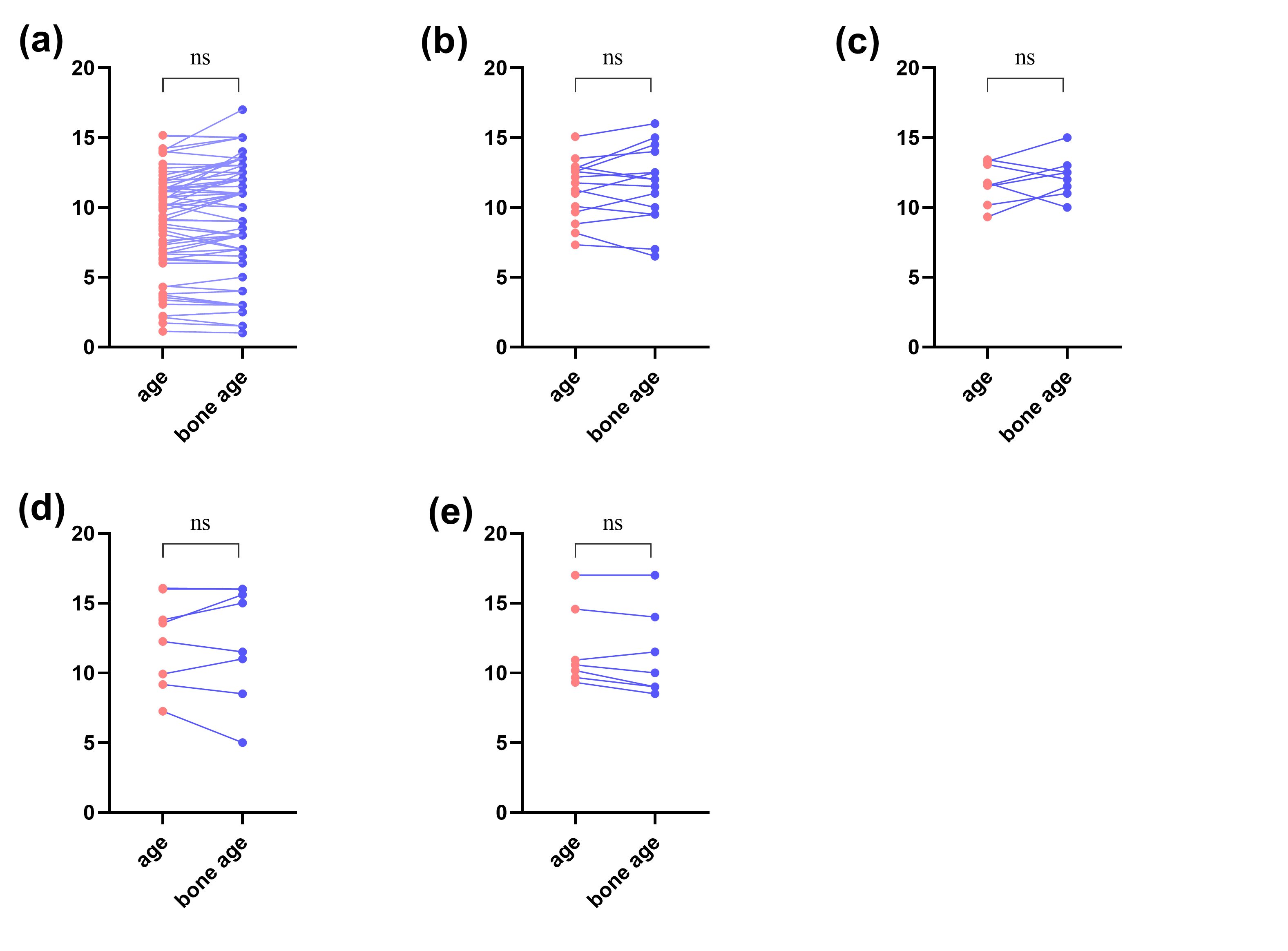

Supplement: Supplementary file 1 — Supplementary Material 1 [file 12887_2024_4740_MOESM1_ESM.tif]

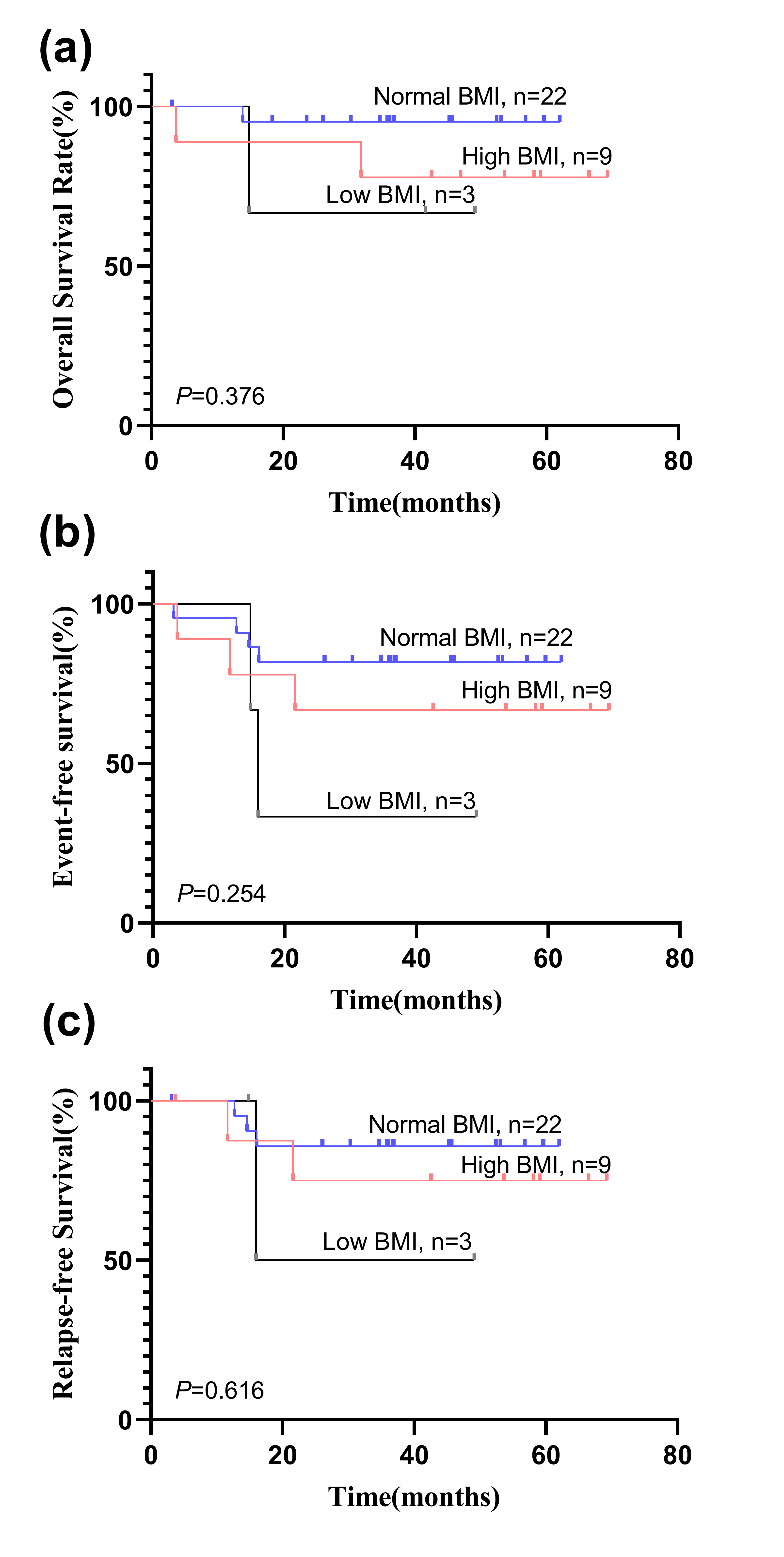

Supplement: Supplementary file 2 — Supplementary Material 2 [file 12887_2024_4740_MOESM2_ESM.tif]

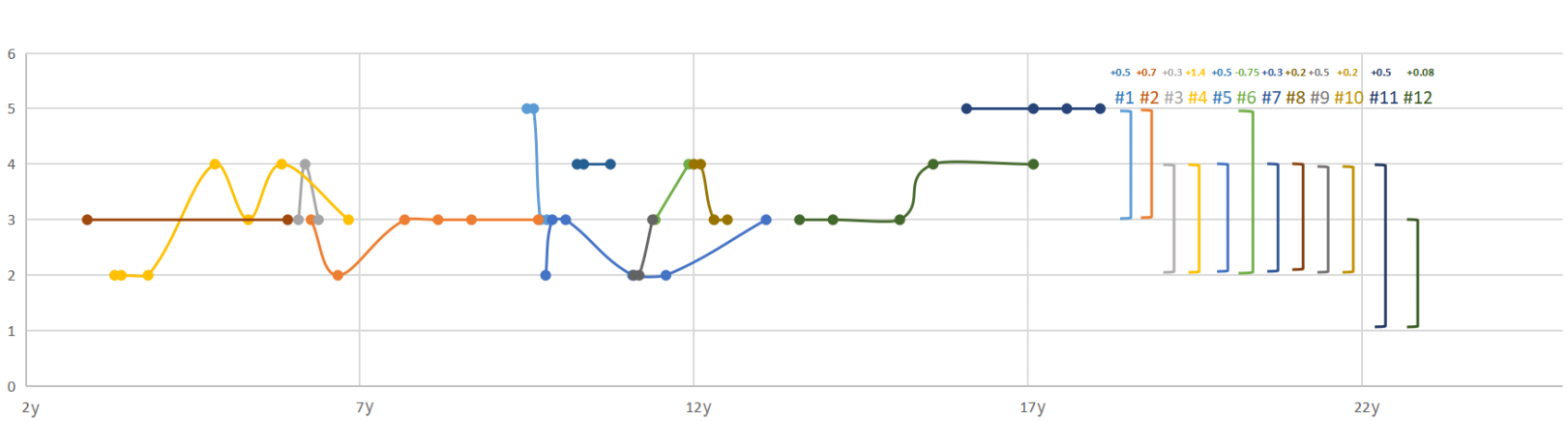

Supplement: Supplementary file 3 — Supplementary Material 3 [file 12887_2024_4740_MOESM3_ESM.tif]
