# Supplementary material for: Longitudinal changes in body mass index, height, and weight in children with acute myeloid leukemia
Source: BMC Pediatr. 2024 Apr 30;24:293. doi: 10.1186/s12887-024-04740-z (PMC11061944; doi:10.1186/s12887-024-04740-z)
Supplement: Supplementary file 4 — Supplementary Material 4 [file 12887_2024_4740_MOESM4_ESM.docx]

Table S1 Clinical characteristics of patients at initial diagnosis between overweight and non-overweight group.

| Clinical characteristic | Overweight group  （n=9） | Non-Overweight group  (n=25) | *P* value |
| --- | --- | --- | --- |
| Sex |  |  | 0.417 |
| Male | 5 (55.56) | 11 (44.00) |  |
| Female | 4 (44.44) | 14 (56.00) |  |
| Age at diagnosis (years) | 9.17 (4.00-11.83) | 7.72 (2.42-13.92) | 0.423 |
| WBC at diagnosis (×10^9^/L) | 23.06 (9-52) | 28.87 (1-138) | 0.598 |
| Fusion transcripts |  |  | 0.366 |
| Without known genes | 1 (11.11) | 8 (32.00) |  |
| *RUNX1-RUNX1T1* | 6 (66.67) | 10 (40.00) |  |
| *KMT2A- KMT2AT3* | 0 (0) | 2 (8.00) |  |
| *KMT2A-others* | 0 (0) | 2 (8.00) |  |
| *CBFβ-MYH11* | 1 (11.11) | 3 (12.00) |  |
| *FUS-ERG* | 0 (0) | 0 (0) |  |
| *NUP98-HOXA9* | 1 (11.11) | 0 (0) |  |
| *KIT* mutation |  |  | 0.281 |
| Positive | 2 (22.22) | 2 (8.00) |  |
| Negative | 7 (77.78) | 23 (92.00) |  |
| Risk rank at initial diagnosis |  |  | 0.956 |
| SR | 5 (55.56) | 15 (60.00) |  |
| MR | 1 (11.11) | 3 (12.00) |  |
| HR | 3 (33.33) | 7 (28.00) |  |

Note: Normal data are given as medians (ranges); qualitative variables are given as numbers (percentages). Overweight was defined as BMI more than 75% at initial diagnosis.

**Figure S1. Patient Outcomes According to BMI Status at new diagnosis.**

3-year overall survival (a), 3-year event-free survival (b) and 3-year relapse-free survival (c) of AML patients according to BMI status at new diagnosis. (a) the 3-year OS of low BMI, normal BMI and high BMI were 66.7±27.2%, 95.2±4.6% and 77.8±13.9% respectively. (b) the 3-year EFS of low BMI, normal BMI and high BMI were 33.3%±27.2, 81.8±8.2% and 66.7±15.7% respectively. (c) the 3-year RFS of low BMI, normal BMI and high BMI were 50.0±35.4%, 85.7±7.6% and 75.0±15.3% respectively. Low BMI was classified if the BMI was <5%, high BMI if the BMI was >75% and 75%≥BMI≥5% belong to normal BMI.

**Figure S2. The difference between age and bone age**

Initial diagnosis, consolidation, and maintenance phases (a), drugs withdraw (b), medication discontinuation at 6 months (c), 1 year (d), and 2 years (e).

**Figure S3. Prediction of twelves patients’ genetic height.**

Note: ^#^ represent each patient. According to height percentile curve for Chinese boys/girls aged 0-18, level 1: 0%~5%; level 2: 6%～25%; level 3: 26%～75%; level 4: 76%～95%; level 5: beyond 95%. Among them last follow-up time, #2 #4 #5 #8 #12 patients were in off 2 years, #11 patient was in off 1 year, #7 #10 patients were in maintain stage, #1 #3 #9 patients were in consolidation stage, #6 patient was in EOI 1 stage. The number above the symbols of #1-#12 stands for BA-CA, which was defined as bone age minus chronological age.
